# Comparative Studies on Thermal, Mechanical, and Flame Retardant Properties of PBT Nanocomposites via Different Oxidation State Phosphorus-Containing Agents Modified Amino-CNTs

**DOI:** 10.3390/nano8020070

**Published:** 2018-01-26

**Authors:** San-E Zhu, Li-Li Wang, Hao Chen, Wei Yang, Anthony Chun-Yin Yuen, Timothy Bo-Yuan Chen, Cheng Luo, Wen-Mei Bi, En-Zhu Hu, Jian Zhang, Jing-Yu Si, Hong-Dian Lu, Kun-Hong Hu, Qing Nian Chan, Guan Heng Yeoh

**Affiliations:** 1 Department of Chemical and Materials Engineering, Hefei University, 99 Jinxiu Avenue, Hefei 230601, China; zhuse@hfuu.edu.cn (S.-E.Z.); wangll@hfuu.edu.cn (L.-L.W.); ch961006@163.com (H.C.); luo828421@163.com (C.L.); mmas418093@163.com (W.-M.B.); huez@hfuu.edu.cn (E.-Z.H.); sijyo@hfuu.edu.cn (J.-Y.S.); hdlu@ustc.edu.cn (H.-D.L.); hukunhong@163.com (K.-H.H.); 2 School of Mechanical and Manufacturing Engineering, University of New South Wales, Sydney NSW 2052, Australia; c.y.yuen@unsw.edu.au (A.C.-Y.Y.); timothy.chen@unsw.edu.au (T.B.-Y.C.); qing.chan@unsw.edu.au (Q.N.C.); g.yeoh@unsw.edu.au (G.H.Y.); 3 Department of Applied Chemistry, Anhui Agricultural of University, Hefei 230036, China

**Keywords:** carbon nanotubes, polymer-matrix nanocomposites, mechanical properties, flame retardancy

## Abstract

High-performance poly(1,4-butylene terephthalate) (PBT) nanocomposites have been developed via the consideration of phosphorus-containing agents and amino-carbon nanotube (A-CNT). One-pot functionalization method has been adopted to prepare functionalized CNTs via the reaction between A-CNT and different oxidation state phosphorus-containing agents, including chlorodiphenylphosphine (DPP-Cl), diphenylphosphinic chloride (DPP(O)-Cl), and diphenyl phosphoryl chloride (DPP(O_3_)-Cl). These functionalized CNTs, DPP(O*_x_*)-A-CNTs (*x* = 0, 1, 3), were, respectively, mixed with PBT to obtain the CNT-based polymer nanocomposites through a melt blending method. Scanning electron microscope observations demonstrated that DPP(O*_x_*)-A-CNT nanoadditives were homogeneously distributed within PBT matrix compared to A-CNT. The incorporation of DPP(O*_x_*)-A-CNT improved the thermal stability of PBT. Moreover, PBT/DPP(O_3_)-A-CNT showed the highest crystallization temperature and tensile strength, due to the superior dispersion and interfacial interactions between DPP(O_3_)-A-CNT and PBT. PBT/DPP(O)-A-CNT exhibited the best flame retardancy resulting from the excellent carbonization effect. The radicals generated from decomposed polymer were effectively trapped by DPP(O)-A-CNT, leading to the reduction of heat release rate, smoke production rate, carbon dioxide and carbon monoxide release during cone calorimeter tests.

## 1. Introduction

Owing to the superior dimensional stability and heat resistance, poly(1,4-butylene terephthalate) (PBT) has been extensively applied in the electrical and electronics industries [1,2]. Nevertheless, PBT resins without flame retarded modification are easily ignitable either by an electric spark or short circuitry within the electrical network which could lead to serious melt-dripping, rapid flame propagation and large production of smoke in an untenable fire condition. With attempts being made to fire-proof PBT, only few nanoadditives, e.g., nano-clay [3,4,5], graphene [6] and polyhedral oligomeric silsesquioxane (POSS) [7], have been explored to improve the flame retardancy of PBT, as well as other properties. Camino et al. [3] reported an organoclay which was prepared by the ion exchange of original alkaline cations with dimethyl hydrogenated tallow benzyl quaternary ammonium chloride. A decrease (66%) of the peak heat release rate (PHRR) values for PBT with the presence of 5% organoclay was obtained. Wang and co-workers [6] prepared MnCo_2_O_4_–graphene (GNS) hybrids, which were then added into the PBT matrix via a masterbatch-melt blending method. The peak heat release rate and smoke production rate values of MnCo_2_O_4_–GNS/PBT composites were decreased by 39.4 and 35.7%, respectively. In our previous study [7], functionalized POSS with phosphorus-containing agent showed significant fire retarded effect in PBT. Apart from the reported flame retardant additives, aluminum phosphinate and aluminum hypophosphite with similar molecular structure and relatively high oxidation state of phosphorus atom have proven to be very effective flame retardants for PBT, due to a combination of gas-phase flame inhibition effect and barrier effect of char layers in the condensed phase [4,8,9].

Carbon nanotubes (CNTs) have attracted enormous attention due to their outstanding mechanical, electrical, and thermal properties [10]. Various applications from nanodevices to nanocomposites have been considered. One promising application of CNTs lies in the development of polymer nanocomposites, since the incorporation of CNTs into polymers at a very low loading can lead to substantial enhancement in the thermal, electrical, mechanical, and flame retardant properties [10,11,12,13]. However, CNTs have poor dispersion characteristics in common solvents and polymeric materials [10,14]. Covalent functionalization of CNTs has been considered as an effective method to overcome the shortcoming of CNTs [10,14,15,16]. For example, CNTs covalently functionalized with pyrrolidine exhibited a solubility of 50 mg/mL in chloroform [15]. CNTs grafted with intumescent flame retardant (PDSPB) promoted the distribution in acrylonitrile-butadiene-styrene copolymer, leading to improved flame retardancy [10]. Functionalization of CNTs with tri(1-hydroxyethyl-3-methylimidazolium chloride) phosphate (IP) promoted the dispersion of CNTs in polylactide (PLA) [16]. The thermal stability and flame retardancy of CNTs/PLA nanocomposites were enhanced according to the literature reports that demonstrated the relationship among dispersion and material properties [17,18]. Consequently, CNTs covalently functionalized with suitable organic modifiers contribute to the improvement of the overall properties of the corresponding polymer nanocomposites.

Although many efforts have been made to achieve the functionalization of CNTs, most of the methods generally involve complex reactions during the treatment process. To the best of our knowledge, a systematic investigation of CNTs functionalization by phosphorus-based flame retardant units with different oxidation state has not been reported. In addition, there is still a great need to purposefully develop a simpler approach to achieve a multi-functional CNTs-based polymer. In reinforcing the flame retardant effect via the use of phosphorus-containing compounds, a simple one-pot functionalization method has been utilized to prepare the covalently functionalized CNT via the reaction between amino-carbon nanotube (A-CNT) and different oxidation state phosphorus-containing agents. In this study, A-CNT reacts with chlorodiphenylphosphine (DPP-Cl,), diphenylphosphinic chloride (DPP(O)-Cl) and diphenyl phosphoryl chloride (DPP(O_3_)-Cl) to prepare the different functionalized CNTs. More importantly, the current study is aimed at fabricating high-performance PBT nanocomposites filled with functionalized CNTs. The dispersion of different functionalized CNTs in PBT matrix is evaluated, and the influence of these functionalized CNTs on the mechanical, thermal and flame retardant properties of the resultant PBT nanocomposites is subsequently assessed.

## 2. Experimental Section

### 2.1. Raw Materials

Poly(1,4-butylene terephthalate) (PBT, B4500) was purchased from BASF Chemical Company, Ludwigshafen, Germany. Amino-carbon nanotube (A-CNT, multi-walled, -NH_2_ content: 0.45 wt %, purity: >95%, length: 10–50 μm, diameter: 8–15 nm) was provided by Chengdu Organic Chemicals Co. Ltd., Chengdu, China. Chlorodiphenylphosphine (DPP-Cl, 97%), diphenylphosphinic chloride (DPP(O)-Cl, 98%), diphenyl phosphoryl chloride (DPP(O_3_)-Cl, 97%), triethylamine (TEA, 99.5%) and *N*,*N*-dimethylformamide (DMF, 99.5%) was obtained from Aladdin Reagent Co. Ltd., Shanghai, China. *N*,*N*-dimethylformamide and triethylamine were dried over 0.4 nm molecular sieves before use. Other reagents were used without further purification.

### 2.2. Preparation of Functionalized CNTs

**Figure 1:**
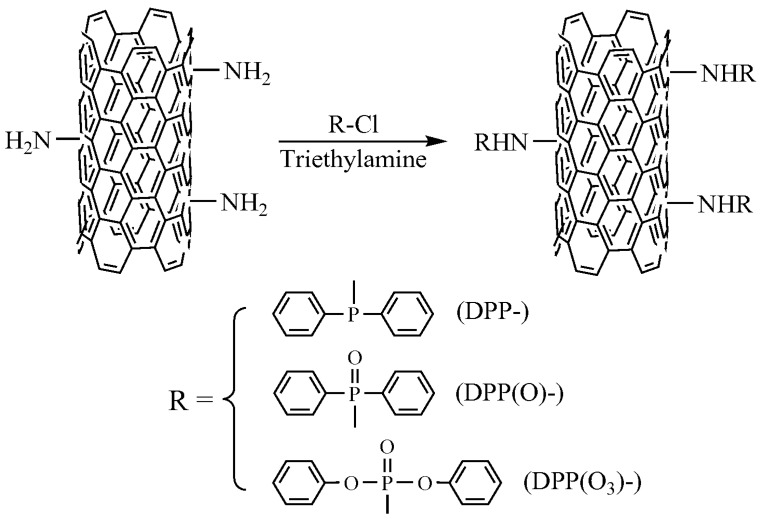
Schematic representation of the preparation route for covalently functionalized CNTs: DPP-A-CNT, DPP(O)-A-CNT, and DPP(O_3_)-A-CNT.

Functionalized CNTs was prepared based on the method reported in our study [19]. Typically, A-CNT (100 mg), DMF (50 mL), and suitable amount of TEA (catalyst) were charged into a three-necked flask. DPP-Cl (75 mg), DPP(O)-Cl (80 mg) and DPP(O_3_)-Cl (90 mg) were then dissolved in DMF (10 mL), and added dropwise into the suspension of A-CNT and TEA. The mixture was stirred in an ice-water bath for 1 h under dry nitrogen condition, subsequently heated up to 80 °C and kept at this temperature for 24 h. The mixture was filtered, washed with DMF and dried in a vacuum oven at 80 °C to a constant weight. These functionalized CNTs are denoted as DPP-A-CNT, DPP(O)-A-CNT and DPP(O_3_)-A-CNT. The preparation route of the functionalized carbon nanotubes is depicted in Figure 1.

### 2.3. Preparation of PBT/Functionalized CNTs Nanocomposites

Before melt processing, PBT, A-CNT, DPP-A-CNT, DPP(O)-A-CNT and DPP(O_3_)-A-CNT were continuously dried at 80 °C for 12 h. In a typical experiment, 0.5 g A-CNT was blended with 49.5 g PBT to prepare the nanocomposites using a torque rheometer (RTOI-55/20, POTOP Co. Ltd., Guangzhou, China) at 235 °C at a constant rotation speed of 100 rpm for 10 min. These mixtures were then molded through a hot press or microinjection molding machine at 240–250 °C to obtain the samples with different sizes for further measurements. Other samples were fabricated according to the same procedure. These nanocomposites were designated as PBT/A-CNT, PBT/DPP-A-CNT, PBT/DPP(O)-A-CNT and PBT/DPP(O_3_)-A-CNT at the same loading of nanoadditives (1 wt %).

### 2.4. Instruments and Measurements

Fourier transform infrared spectroscopy (FTIR) spectra were recorded on a FTIR spectrophotometer (Nicolet 6700, Thermo Fisher Scientific Inc., Waltham, MA, USA). Raman spectra were obtained from a DXR Smart Raman Spectrometer (Thermo Fisher Scientific Inc.), in the wavenumber range of 500–2000 cm^−1^. Thermal decomposition behaviors of samples under nitrogen atmosphere were investigated by a Q5000 IR TGA (TA Instruments, New Castle, DE, USA) from 25 to 700 °C at a heating rate of 20 °C/min. The dispersion and morphology of nano-additives were observed via scanning electron microscope (SEM) on a Hitachi SU8010 SEM (Tokyo, Japan) with the acceleration voltage of 10 kV. The samples were obtained by immersing PBT nanocomposites into liquid nitrogen, and a conductive gold layer was coated on the fractured surface prior to SEM observations.

Thermal behaviors were studied by a Perkin Elmer Diamond DSC under nitrogen condition. Samples were heated from 50 to 300 °C at a heating rate of 10 °C/min and kept at 300 °C for 5 min to eliminate any thermal history, and they were then cooled to 50 °C at a rate of 10 °C/min. Finally, these samples were maintained at 50 °C for 5 min and heated to 300 °C. They were held at 300 °C for 5 min, and subsequently cooled to 50 °C at 10 °C/min.

Tensile properties were evaluated by a WD-20D universal testing machine according to the standard ASTM D-638. The width and thickness of specimens were 4.0 ± 0.1 mm and 2.0 ± 0.1 mm, respectively. The crosshead speed was set as 20 mm/min. Five runs for each sample were measured, and the average value was recorded.

Flame retardant properties were investigated on a FTT cone calorimeter (FTT, Derby, UK) based on the ISO 5660-1 standard. The sample size was 100 mm × 100 mm × 3.0 mm. All samples were wrapped by a layer of aluminum foil, and they were then irradiated under a heat flux of 35 kW/m^2^. Residues were analyzed by SEM coupled with energy dispersive X-ray (EDX). The surface elements were attained from EDX on an EMAX energy spectroscopy (HORIBA, Ltd., Kyoto, Japan).

## 3. Results and Discussion

### 3.1. Characterization of Functionalized CNTs

**Figure 2:**
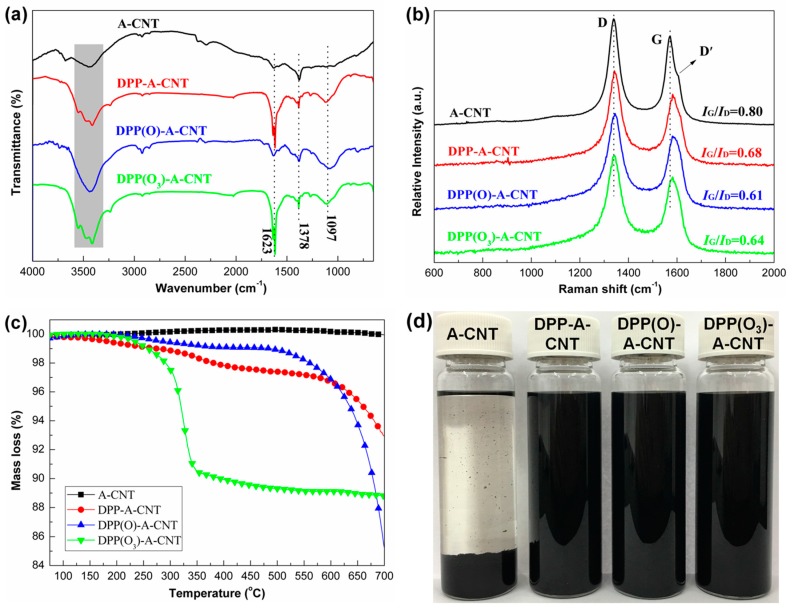
Characterizations of A-CNT, DPP-A-CNT, DPP(O)-A-CNT and DPP(O_3_)-A-CNT: (**a**) FTIR spectra; (**b**) Raman spectra; (**c**) thermogravimetric curves; and (**d**) the dispersion of functionalized CNTs in DMF after one week.

Figure 2a shows the FTIR spectra of A-CNT, DPP-A-CNT, DPP(O)-A-CNT and DPP(O_3_)-A-CNT. The bands at 3425 and 1630 cm^−1^ are a result of the N-H stretching and bending vibrations, respectively. The peak at 1378 cm^−1^ is due to the stretching vibrations of C–N. The strong characteristic peak at 1097 cm^−1^ in the FTIR spectra of DPP-A-CNT, DPP(O)-A-CNT and DPP(O_3_)-A-CNT corresponds to P–N stretching vibration [11,20]. The results demonstrate that DPP-Cl, DPP(O)-Cl and DPP(O_3_)-Cl are reacted with amino groups in A-CNT through nucleophilic substitution, leading to the graft modification of phosphorus-nitrogen containing compounds onto the surface of A-CNT.

The Raman spectra of A-CNT, DPP-A-CNT, DPP(O)-A-CNT and DPP(O_3_)-A-CNT are illustrated in Figure 2b. Two main absorption peaks at 1340 and 1571 cm^−1^ are attributed to the D-band and G-band, respectively [21]. The G-band is attributed to the first-order scattering of the sp^2^ carbon atoms of CNTs, while the D-band corresponds to the disorder-induced or sp^3^ carbon atoms of CNTs [22,23,24]. A new band D’ at higher wavenumber close to G-band corresponds to the functionalized CNTs [24,25]. All the G-bands in Raman spectra of DPP-A-CNT, DPP(O)-A-CNT and DPP(O_3_)-A-CNT indicate a spectral shift compared to A-CNT, implying sidewall or end-cap modification [25]. The relative intensity ratios of G-band to D-band (*I*_G_/*I*_D_), which evaluate the purity and functionalization of CNTs, are 0.80 for A-CNT, 0.68 for DPP-A-CNT, 0.61 for DPP(O)-A-CNT, and 0.64 for DPP(O_3_)-A-CNT. The decreased *I*_G_/*I*_D_ values demonstrate the successful grafting of A-CNT with different oxidation state phosphorus-containing compounds.

The thermogravimetric curves of A-CNT, DPP-A-CNT, DPP(O)-A-CNT and DPP(O_3_)-A-CNT under nitrogen atmosphere are shown in Figure 2c. A-CNT depicts no obvious mass loss in the whole temperature range studied. DPP-A-CNT, DPP(O)-A-CNT and DPP(O_3_)-A-CNT exhibits approximately 7.1%, 14.7% and 11.2% mass loss at 700 °C, respectively, which can be attributed to the decomposition of functionalized species on the A-CNT.

To evaluate the dispersion of functionalized CNTs, the mixtures of CNTs and functionalized CNTs in DMF (2 mg/mL) was treated by ultrasonication for 5 min. Digital photographs of the dispersion state of A-CNT, DPP-A-CNT, DPP(O)-A-CNT and DPP(O_3_)-A-CNT mixtures after a week are represented in Figure 2d. A-CNT can be seen to exhibit poor dispersion characteristic in DMF with a solid precipitate, while DPP-A-CNT, DPP(O)-A-CNT and DPP(O_3_)-A-CNT form stable DMF mixtures. Because DMF is a good solvent for DPP-Cl, DPP(O)-Cl and DPP(O_3_)-Cl, functionalized CNTs grafted with these organic species clearly demonstrated better dispersion characteristic in DMF, which is consistent with the reported results [10,19].

### 3.2. Morphological Analysis

**Figure 3:**
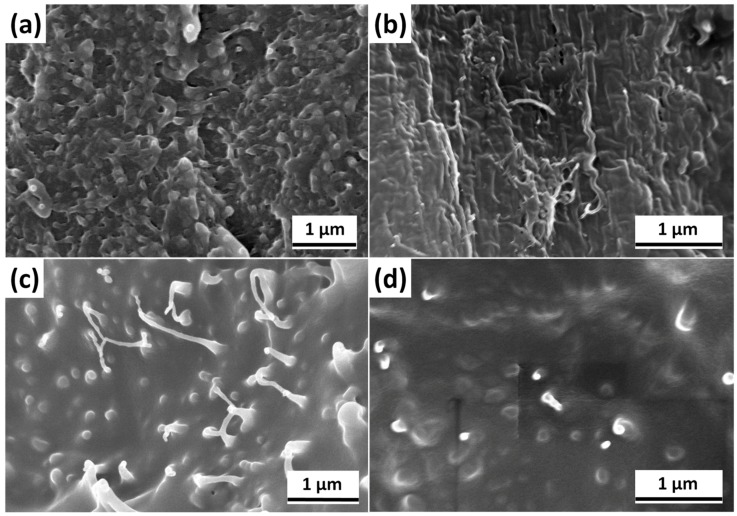
Scanning electron microscope (SEM) images of the composite fracture surfaces: (**a**) PBT/A-CNT; (**b**) PBT/DPP-A-CNT; (**c**) PBT/DPP(O)-A-CNT; and (**d**) PBT/DPP(O_3_)-A-CNT.

Cross-sections of PBT/A-CNT, PBT/DPP-A-CNT, PBT/DPP(O)-A-CNT and PBT/DPP(O_3_)-A-CNT nanocomposites characterized by SEM are illustrated in Figure 3. It can be observed that A-CNT is poorly distributed in the PBT matrix with agglomerated nanoparticles. In general, the amino groups on the surface of the A-CNT have the propensity of enhancing the interaction between PBT and A-CNT through the hydrogen bonding formation. However, A-CNTs could not be well distributed in PBT, due to low functionalization. Compared to A-CNTs, DPP-A-CNT, DPP(O)-A-CNT and DPP(O_3_)-A-CNT nanoparticles are homogeneously dispersed in the PBT matrix, as shown in Figure 3b–d. The functional organic groups on the surface of DPP(O*_x_*)-A-CNT (*x* = 0, 1, 3) inhibit the agglomeration of A-CNTs [26]. Although the dispersion of DPP-A-CNT nanoparticles in PBT is improved, the interfacial adhesion between DPP-A-CNT and PBT remains weak, which is reflected by the presence of an interface. On the contrary, DPP(O)-A-CNT and DPP(O_3_)-A-CNT nanoparticles are well integrated with the PBT molecular chains, leading to the absence of the interface between functionalized CNTs and PBT. The PBT macromolecular chains probably would have enveloped the DPP(O)-A-CNT and DPP(O_3_)-A-CNT nanoparticles, resulting in the formation of non-covalent crosslinking points [27,28,29].

**Figure 4:**
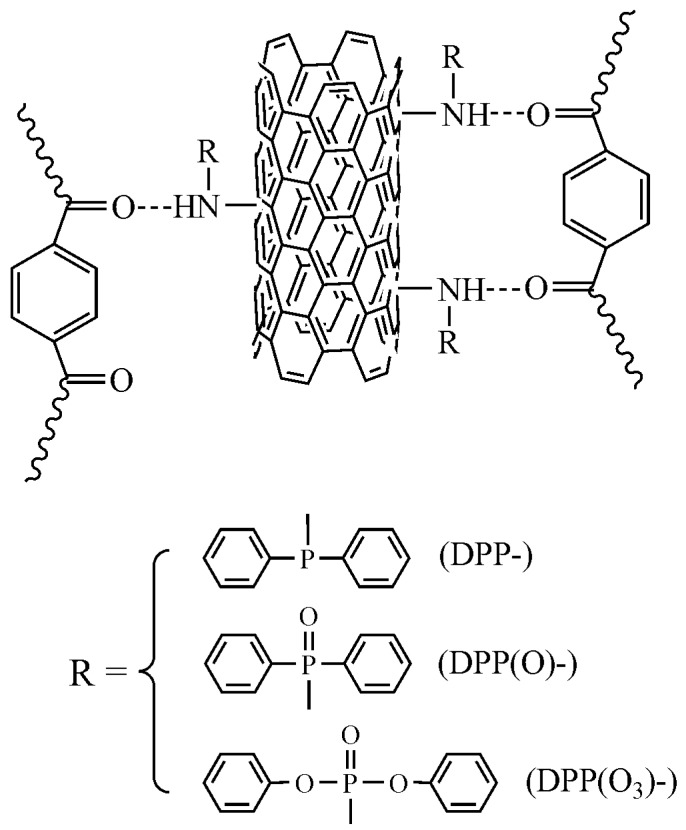
Schematic representation of the hydrogen-bond interaction between functionalized CNTs and PBT macromolecular chains.

Figure 4 shows the schematic pattern of the hydrogen-bond interaction between DPP(O*_x_*)-A-CNT and PBT. Because the electronegativity of nitrogen (N) atom is higher than that of phosphorus (P) atom, the electronic density around N atom in DPP-A-CNT increases, leading to the reduction of electropositivity of hydrogen (H) atom in secondary amine. Therefore, the hydrogen-bond interaction between DPP-A-CNT and PBT is weak. As the quantity of oxygen atom increases, the electronic density in N atom decreases in DPP(O)-A-CNT and DPP(O_3_)-A-CNT, resulting in the enhancement of electropositivity of H atom in secondary amine. As a result, the hydrogen-bond interaction between DPP(O_3_)-A-CNT and PBT is highest. Owing to the strong hydrogen-bond interaction, the PBT macromolecular chains can firmly envelope the DPP(O)-A-CNT and DPP(O_3_)-A-CNT nanoparticles. Because of the poor dispersion characteristic of A-CNT in PBT, the material properties of PBT/A-CNT will not be evaluated in the current study.

### 3.3. Thermal Properties

**Figure 5:**
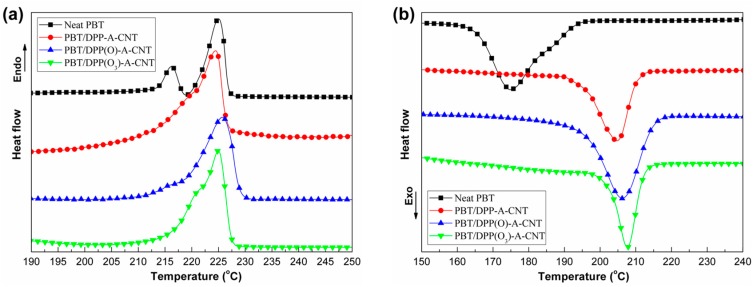
DSC curves of: heating scan (**a**); and non-isothermal crystallization (**b**) at a rate of 10 °C/min for neat PBT, PBT/DPP-A-CNT, PBT/DPP(O)-A-CNT and PBT/DPP(O_3_)-A-CNT.

The melt and non-isothermal crystallization behaviors of neat PBT and PBT/DPP(O*_x_*)-A-CNT nanocomposites were characterized by DSC. Figure 5 depicts the thermal behavior curves recorded for all samples at the heating and cooling scan of 10 °C/min. The thermal parameters obtained from the thermograms are summarized in Table 1. In the heating scan, the influence of DPP(O*_x_*)-A-CNT on melting temperature (*T*_m_) of PBT can be seen to be negligible. The multiple melt behavior observed for neat PBT (Figure 5a) is caused by the fusion of a certain amount of original crystals, followed by the recrystallization and final melting of more perfect crystals, partly formed during primary crystallization and through the recrystallization process occurring during the heating scan [30,31,32]. However, the overlapping of two peaks of PBT mix forms a new peak with the introduction of DPP(O*_x_*)-A-CNT nanoparticles. This indicates that the presence of DPP(O*_x_*)-A-CNT nanoparticles improve the crystallization process, leading to the formation of more perfect and stable crystals, which becomes melt at higher temperature [32,33,34]. Therefore, the first melting peak shifts to high temperature region, resulting in the reduction of multiple melt behavior.

As shown in Figure 5b, all crystallization temperatures (*T*_c_) of the PBT nanocomposites are significantly improved with the addition of DPP(O*_x_*)-A-CNT nanoparticles. In comparison with that of neat PBT, the *T*_c_ value of PBT/DPP(O_3_)-A-CNT evaluated by DSC is increased by 34 °C, higher than that of PBT/carboxylated-CNT reported in our previous work [9,35]. Functionalized CNTs show more significant heterogeneous nucleation effect on the crystallization process of PBT. On the other hand, the *T*_c_ value of the three nanocomposites increases with the increasing quantity of oxygen atom, due to the different hydrogen-bond interaction: (PBT/DPP(O_3_)-A-CNT > PBT/DPP(O)-A-CNT > PBT/DPP-A-CNT). The strong hydrogen-bond interaction promotes the PBT macromolecular chains to envelope the CNT nanoparticles thereby resulting in the formation of non-covalent crosslinking points, which can strongly restrict the segmental motion of PBT chains [27,28,29], thus accelerating its crystallization process. When the crystallization begins at higher temperature, more perfect and stable crystals will be formed, which is beneficial to the improvement of mechanical strength as well as the reduction of multiple melt behavior.

### 3.4. Thermal Decomposition Behaviors

**Figure 6:**
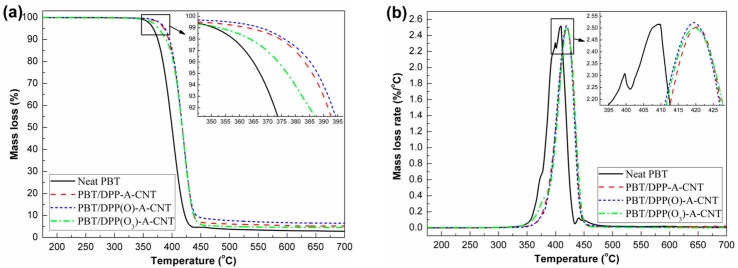
Thermal decomposition curves of neat PBT, PBT/DPP-A-CNT, PBT/DPP(O)-A-CNT and PBT/DPP(O_3_)-A-CNT under nitrogen condition: (**a**) mass loss; and (**b**) mass loss rate.

The thermal decomposition behaviors of neat PBT and its nanocomposites under nitrogen condition are shown in Figure 6, and the corresponding data are summarized in Table 2. In Figure 6a, it is seen that the thermal decomposition behavior of each sample exhibits a one-stage degradation process. Neat PBT leaves only 2.7 wt % char residue at 700 °C. In the thermal decomposition process, the main volatiles, composed of butadiene, carbon dioxide, tetrahydrofuran, benzoic acid and ester derivatives, are released, leaving small solid residues with acidic and anhydride structures [8,9]. The addition of DPP(O*_x_*)-A-CNT nanoparticles results in the improvement of thermal stability and char yields of PBT, and PBT/DPP(O)-A-CNT is seen to be more efficient than the other two functionalized CNTs. For example, the *T*_−5%_ value is increased from 367 °C for neat PBT to 388 °C for PBT/DPP(O)-A-CNT, and the *T*_max_ value is improved from 408 to 419 °C. PBT/DPP(O_3_)-A-CNT shows inferior thermal stability due to the unstable organic phosphate grafted on the surface of DPP(O_3_)-A-CNT, while the thermal stability is still higher than that of neat PBT. The results show that the presence of DPP(O*_x_*)-A-CNT nanoparticles can significantly enhance the thermal stability of the PBT nanocomposites, which aligns with findings in our previous studies [9,35]. The improved thermal stability is attributed to the excellent thermal conductivity and homogeneous dispersion of functionalized CNTs [36,37].

PBT/DPP(O)-A-CNT has a high residual weight (6.5 wt %), more than that of PBT/DPP-A-CNT (5.2 wt %) and PBT/DPP(O_3_)-A-CNT (4.5 wt %), as shown in Figure 6a and Table 2. DPP(O_3_)-A-CNT with high oxidation state phosphorus-containing groups shows weak carbonization effect in PBT during the thermal decomposition. The phosphorus-containing compound with low oxidation state may preferably promote the cross-linking reaction between the organophosphorus-based pyrolysis products and ester derivatives decomposed from PBT chains. From the SEM observations of PBT/DPP(O*_x_*)-A-CNT fracture surfaces, it is known that the PBT macromolecular chains compactly envelope the DPP(O)-A-CNT nanoparticles due to the strong hydrogen-bond interaction, which is beneficial for cross-linking reaction. In combination with the barrier effect of CNTs, more organic phosphate-based derivatives are formed in the condensed phase, enhancing the strength and thermal stability of the char layer. Figure 6b reveals that the addition of DPP(O*_x_*)-A-CNT nanoparticles improve the *T*_max_ value of PBT, but there is no substantial influence on the maximum mass loss rates (MMLR). These results demonstrate that the introduction of DPP(O*_x_*)-A-CNT can improve the thermal stability and char yields, but does not alter the decomposition pathway of PBT.

### 3.5. Tensile Properties

**Figure 7:**
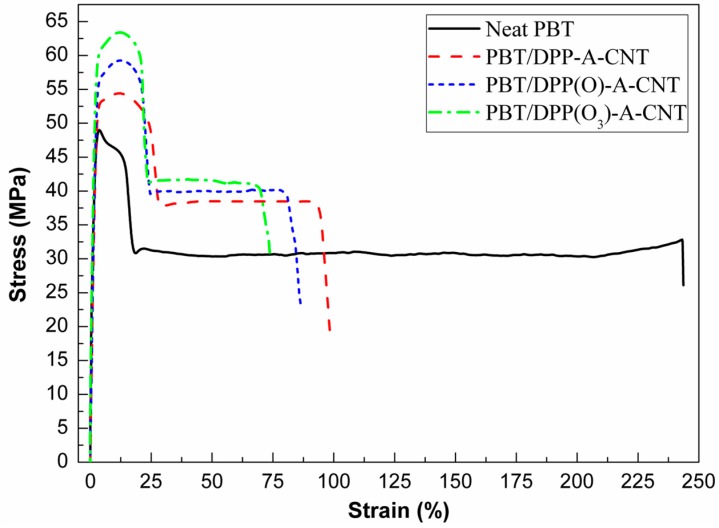
Tensile stress-strain curves of neat PBT, PBT/DPP-A-CNT, PBT/DPP(O)-A-CNT and PBT/DPP(O_3_)-A-CNT.

The tensile properties of neat PBT and its nanocomposites are illustrated in Figure 7. The corresponding data are summarized in Table 3. From the stress-strain curves, it is seen that the introduction of the three DPP(O*_x_*)-A-CNT nanoparticles into PBT improves the tensile strength due to nano-reinforcing effect of CNTs with ultra-high aspect surface area [27,38]. PBT/DPP(O_3_)-A-CNT shows the highest tensile strength of 62.1 MPa (PBT/DPP(O_3_)-A-CNT > PBT/DPP(O)-A-CNT > PBT/DPP-A-CNT) which is 25% enhancement relative to neat PBT. The enhanced tensile properties of PBT/DPP(O_3_)-A-CNT nanocomposites is attributed to better interfacial bonding occurring between DPP(O_3_)-A-CNT and PBT matrix as well as better dispersion of DPP(O_3_)-A-CNT, compared to DPP-A-CNT and DPP(O)-A-CNT. Morphological analysis reveals that DPP(O_3_)-A-CNT shows the best dispersion characteristic in PBT, and the PBT macromolecular chains envelope the DPP(O_3_)-A-CNT nanoparticles forming strong interfacial adhesion. The strong interfacial adhesion, resulting from the hydrogen-bond interaction (Figure 4), is favorable to load transfer from the polymer matrix to the CNTs. As shown in Figure 7 and Table 3, the elongation at break for the PBT nanocomposites decreases with the introduction of DPP(O*_x_*)-A-CNT nanoparticles (PBT/DPP-A-CNT > PBT/DPP(O)-A-CNT > PBT/DPP(O_3_)-A-CNT), while the tensile strength values show the opposite trend. PBT nanocomposites become brittle in comparison with neat PBT, because of the increased stiffness of the PBT nanocomposites and the micro-voids formed around the nanotubes during the tensile measurement [27,39]. The elongation at break for PBT nanocomposites is in relation with the interfacial interaction between CNTs and PBT matrix. The stronger interfacial adhesion, the more difficult segmental stretching and motion of PBT chains, the lower elongation at break [39].

### 3.6. Flame Retardancy

**Figure 8:**
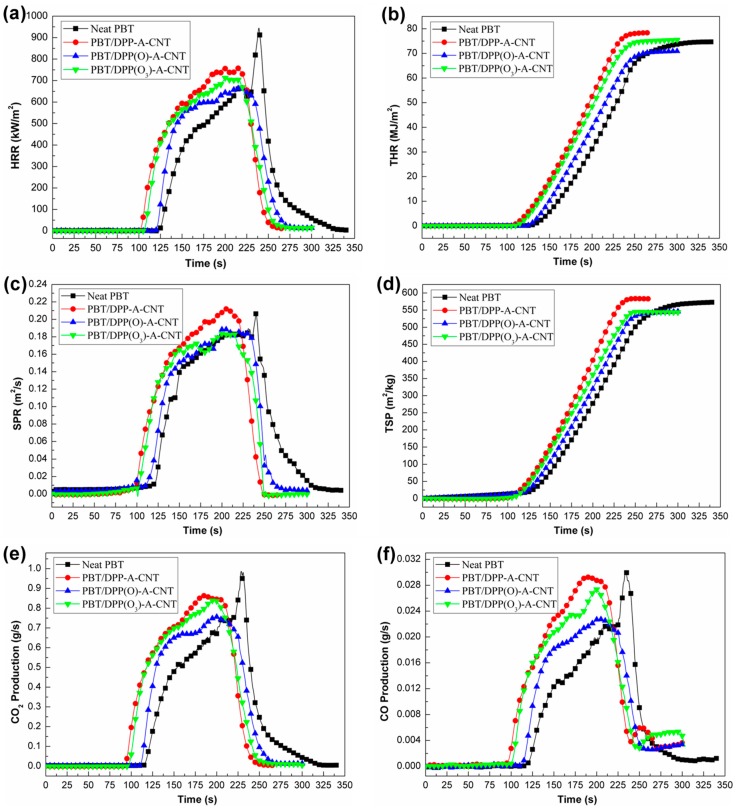
(**a**) heat release rate (HRR); (**b**) total heat release (THR); (**c**) smoke production rate (SPR); (**d**) total smoke production (TSP); (**e**) CO_2_ production; and (**f**) CO production as a function of the burning time for neat PBT, PBT/DPP-A-CNT, PBT/DPP(O)-A-CNT, and PBT/DPP(O_3_)-A-CNT in the cone calorimeter tests at 35 kW/m^2^.

Cone calorimeter tests [40,41] were performed to measure the heat release rate (HRR), total heat release (THR), smoke production rate (SPR), and CO_2_ and CO productions of PBT and its nanocomposites. The HRR and THR curves under a heat flux of 35 kW/m^2^ are shown in Figure 8a,b, respectively, and the related data are listed in Table 4. Neat PBT burns at 120 s (TTI value) with a high PHRR value (944 kW/m^2^). The incorporation of DPP(O*_x_*)-A-CNT nanoparticles lead to the slightly reduced TTI values and increased full width at half maximum for the HRR curves. The PHRR value decreases from 944 kW/m^2^ for neat PBT to 759 kW/m^2^ for PBT/DPP-A-CNT, 668 kW/m^2^ for PBT/DPP(O)-A-CNT, and 710 kW/m^2^ for PBT/DPP(O_3_)-A-CNT, with reductions of 20%, 29%, and 25%, respectively. From the THR curves shown in Figure 8b, it can be observed that PBT/DPP(O)-A-CNT demonstrates the lowest THR value, which is reduced by 5% compared to neat PBT. The results show that the introduction of DPP(O*_x_*)-A-CNT nanoparticles inhibits the heat release through the barrier effect of the char residues and nanoparticle networks, of which DPP(O)-A-CNT exhibits the highest reduction on HRR and THR.

PBT is a kind of aromatic polymers which releases lots of smoke and toxic gases during burning process [42,43,44]. The reduction of smoke and toxic products during the combustion process is a very important consideration in view of the tenability condition for occupants in enclosed environments. Figure 8c,d exhibit the SPR and total smoke production (TSP) curves of neat PBT and its nanocomposites. The corresponding data are summarized in Table 4. Neat PBT shows high peak SPR (PSPR) and TSP values. The presence of DPP-A-CNT results in the occurrence of wide and flat SPR curve. However, the PSPR and TSP values of PBT/DPP-A-CNT are slightly increased. The incorporation of DPP(O)-A-CNT and DPP(O_3_)-A-CNT reduces the PSPR value. It decreases from 0.207 m^2^/s for neat PBT to 0.189 m^2^/s for PBT/DPP(O)-A-CNT and 0.183 m^2^/s for PBT/DPP(O_3_)-A-CNT with reductions of 9% and 12%, respectively. The TSP values of PBT are also reduced by the addition of DPP(O)-A-CNT and DPP(O_3_)-A-CNT. The results indicate that the introduction of DPP(O)-A-CNT and DPP(O_3_)-A-CNT effectively inhibit the smoke production during the combustion process of PBT nanocomposites.

**Figure 9:**
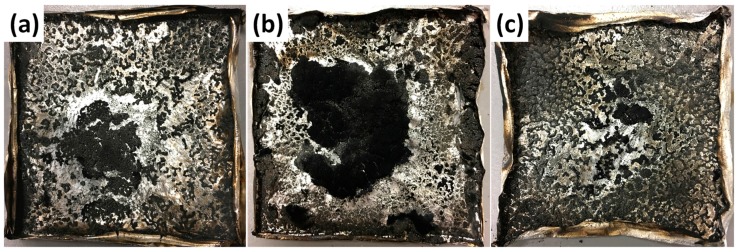
Digital photographs of the residues after cone calorimeter tests: (**a**) PBT/DPP-A-CNT; (**b**) PBT/DPP(O)-A-CNT; and (**c**) PBT/DPP(O_3_)-A-CNT.

In a fire scenario, CO and CO_2_ are the main toxic gases generated from the burning of polymers [45,46,47], which can lead to asphyxiation or suffocation of occupants in enclosed environments. Figure 8e,f represents the CO_2_ and CO production curves of pure PBT and its nanocomposites. The related parameters are listed in Table 4. The addition of DPP(O*_x_*)-A-CNT nanoparticles into PBT brings a reduction in PCO_2_P and PCOP. PBT/DPP(O)-A-CNT exhibits more significant inhibition effect in the emission of CO and CO_2_. Compared to pure PBT, the PCO_2_P and PCOP values for PBT/DPP(O)-A-CNT are reduced by nearly 23% and 24%, respectively. The enhanced fire retarded properties of PBT/DPP(O*_x_*)-A-CNT nanocomposites are primarily attributed to the carbonization effect. From Figure 9, it is clearly seen that the highest char yields for PBT/DPP(O)-A-CNT are left after cone calorimeter tests. Results of TGA indicate that DPP(O)-A-CNT shows more outstanding carbonization effect on PBT, which is consistent with the higher residual yield for PBT/ DPP(O)-A-CNT (7.4 wt %) in cone calorimeter tests. Hence, the incorporation of DPP(O)-A-CNT particles can preferably promote the carbonization of PBT matrix during the combustion process.

**Figure 10:**
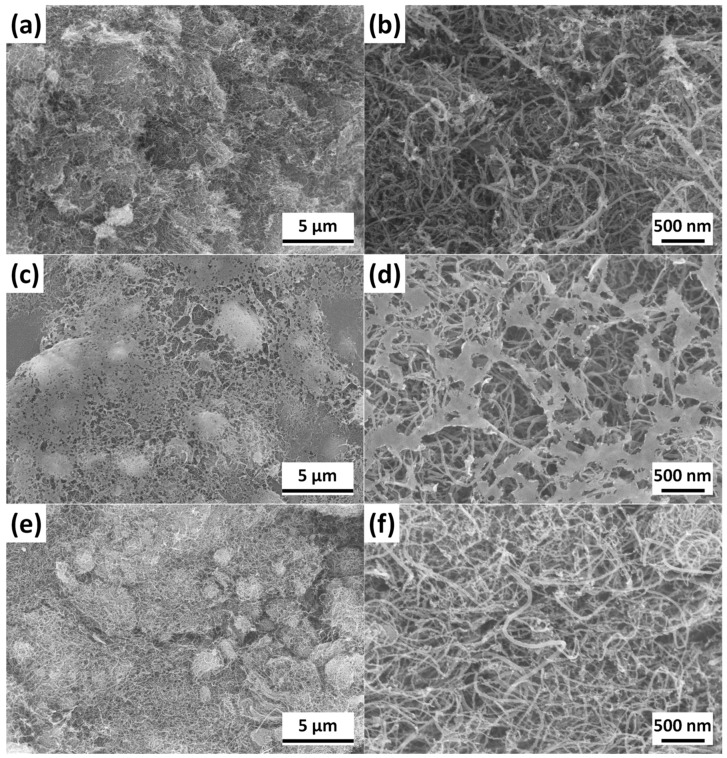
SEM images of the residues after cone calorimeter tests: (**a**,**b**) PBT/DPP-A-CNT; (**c**,**d**) PBT/DPP(O)-A-CNT; and (**e**,**f**) PBT/DPP(O_3_)-A-CNT.

**Figure 11:**
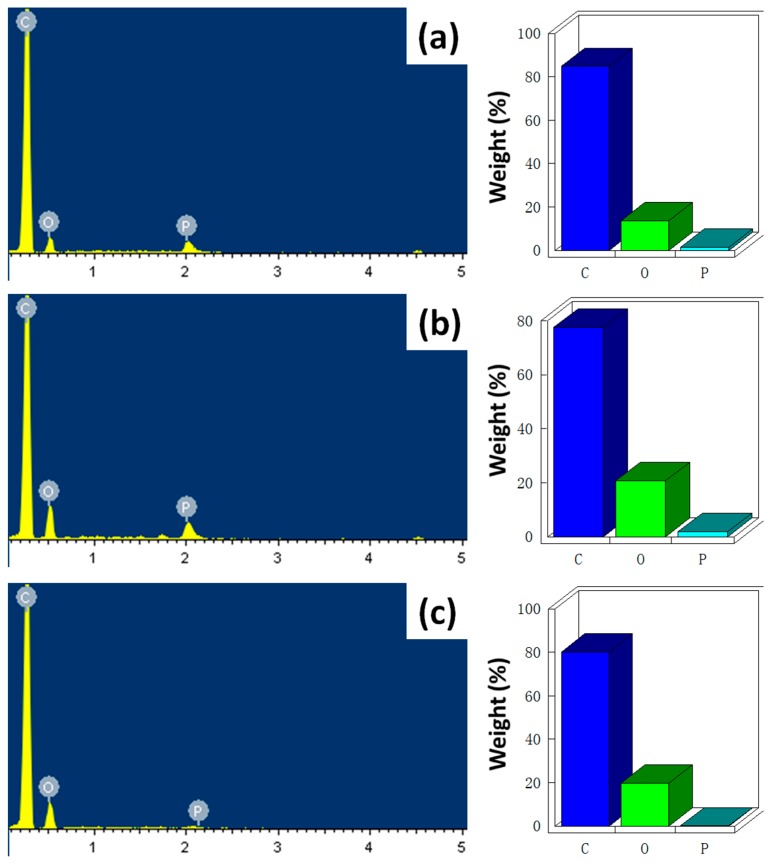
EDX chemical compositions of the residues after cone calorimeter tests: (**a**) PBT/DPP-A-CNT; (**b**) PBT/DPP(O)-A-CNT; and (**c**) PBT/DPP(O_3_)-A-CNT.

To investigate the flame retardant mechanism, the structures of these residues were characterized by SEM coupled with EDX analyzer. The SEM images of the residues for PBT/DPP(O*_x_*)-A-CNT nanocomposites are shown in Figure 10. From the SEM images, there appeared to be more solid chars left behind on the surface of PBT/DPP(O)-A-CNT, compared to PBT/DPP-A-CNT and PBT/DPP(O_3_)-A-CNT. This could be explained by the better carbonization effect of DPP(O)-A-CNT on PBT matrix, which corresponds well with the TGA analysis and residue results from cone calorimeter tests. The EDX results of the residues for PBT/DPP(O*_x_*)-A-CNT nanocomposites are shown in Figure 11, and the related data are summarized in Table 5. All the residue samples are primarily composed of C, O, and P element (element content: C > O > P). In the case of PBT/DPP(O_3_)-A-CNT, the proportion of P element is only 0.13%. The phosphorus-containing groups decomposed from DPP(O_3_)-A-CNT participates in the gas-phase flame retardant action. Compared to PBT/DPP-A-CNT, more O and P elements are left in the char layers of PBT/DPP(O)-A-CNT. The improved O and P elements contents contribute to the reduction of HRR, CO_2_ and CO production as well as smoke emission. Moreover, the phosphinic-based groups decomposed from DPP(O)-A-CNT may efficiently trap radicals to participate in the carbonization reaction. The thermally stable chars act as an effective barrier to reduce the exposure of PBT nanocomposites to an external heat source [48,49,50,51,52,53,54], which is beneficial to reduce the fire hazards.

## 4. Conclusions

With the aim of improving the fire performance of PBT, three covalently functionalized CNTs, DPP(O*_x_*)-A-CNTs (*x* = 0, 1, 3), were successfully prepared and mixed with PBT using one-pot functionalization method via the reaction between different oxidation state phosphorus-containing agents and amino-carbon nanotube (A-CNT). These covalently functionalized CNTs were embedded with PBT through the consideration of a melt blending method. SEM observations revealed that the DPP(O*_x_*)-A-CNT nano-fillers were found to be more homogeneously distributed within the PBT matrix compared with A-CNT alone. The incorporation of the three DPP(O*_x_*)-A-CNT nanoparticles significantly improved the thermal stability of PBT. PBT/DPP(O_3_)-A-CNT showed the highest crystallization temperature and tensile strength, resulting from the good dispersion and interfacial interactions between DPP(O_3_)-A-CNT and PBT matrix. PBT/DPP(O)-A-CNT exhibited the best flame retardant properties due to the excellent carbonization effect. The decomposed polymer radicals can be effectively trapped by DPP(O)-A-CNT, leading to the reduction of PHRR, SPR, PCO_2_P and PCOP in cone calorimeter tests. This simple method to prepare functionalized CNTs in the current work can be extended to the surface functionalization of other nanoadditives. Functionalized nano-additives will enhance the dispersion and interfacial interaction within polymer hosts, resulting in the superior properties of polymeric materials, which shows the promising industrial application.

## Figures and Tables

**Table 1:** Calorimetric data of the melting and non-isothermal crystallization processes for each sample (*T*_m_, melting peak temperature of 2nd heating; *T*_c_, crystallization peak temperature of 2nd cooling).

Sample No.	*T*_m_ (°C)	*T*_c_ (°C)
Neat PBT	225	174
PBT/DPP-A-CNT	224	205
PBT/DPP(O)-A-CNT	226	206
PBT/DPP(O_3_)-A-CNT	225	208

**Table 2:** TGA data under nitrogen condition of each sample. (20 °C/min, 5–10 mg; errors ± 0.5 wt %, ±1 °C).

Sample No.	*T*_−1%_ (°C)	*T*_−5%_ (°C)	*T*_max_ (°C)	Residue at 700 °C (wt %)
Neat PBT	351	365	410	2.9
PBT/DPP-A-CNT	362	386	420	5.2
PBT/DPP(O)-A-CNT	366	388	419	6.5
PBT/DPP(O_3_)-A-CNT	352	377	419	4.5

**Table 3:** Tensile properties for each sample.

Sample No.	Tensile Strength (MPa)	Elongation at Break (%)
Neat PBT	49.5 ± 2.1	244 ± 10
PBT/DPP-A-CNT	55.1 ± 1.9	99 ± 15
PBT/DPP(O)-A-CNT	58.5 ± 1.8	86 ± 12
PBT/DPP(O_3_)-A-CNT	62.1 ± 2.7	74 ± 11

**Table 4:** Cone calorimeter data for each sample at 35 kW/m^2^. (TTI: time to ignition; PHRR: peak heat release rate; THR: total heat release; PSPR: peak smoke production rate; TSP: total smoke production; PCO_2_P: peak CO_2_ production; PCOP: peak CO production).

Sample No.	TTI (s)	PHRR (kW/m^2^)	THR (MJ/m^2^)	PSPR (m^2^/s)	TSP (m^2^/kg)	PCO_2_P (g/s)	PCOP (g/s)	Residue (wt %)
Error	±2	±15	±0.5	±0.01	±20	±0.02	±0.005	±0.2
Neat PBT	120	944	74.7	0.207	573	0.987	0.0300	2.9
PBT/DPP-A-CNT	101	759	78.4	0.213	583	0.866	0.0293	5.6
PBT/DPP(O)-A-CNT	117	668	70.9	0.189	546	0.760	0.0228	7.4
PBT/DPP(O_3_)-A-CNT	104	710	75.5	0.183	544	0.838	0.0273	4.8

**Table 5:** Energy dispersive X-ray (EDX) data of the residues for PBT nanocomposites after cone calorimeter tests.

Sample No.	Element Content (wt %)		
	C	O	P
PBT/DPP-A-CNT	84.82	13.58	1.60
PBT/DPP(O)-A-CNT	77.51	20.63	1.86
PBT/DPP(O_3_)-A-CNT	80.17	19.70	0.13
